# Consensus Panel Recommendations for Optimizing Use of a Contact‐Cooled 1726 nm Laser for the Treatment of Acne

**DOI:** 10.1111/jocd.70699

**Published:** 2026-01-29

**Authors:** Andrei Metelitsa, R. Sonia Batra, Anne Chapas, Jeffrey Fromowitz, Emmy Graber

**Affiliations:** ^1^ University of Calgary Calgary Canada; ^2^ Beacon Dermatology Calgary Canada; ^3^ Batra Dermatology Santa Monica California USA; ^4^ USC Keck School of Medicine Los Angeles California USA; ^5^ UnionDerm New York New York USA; ^6^ Department of Dermatology Mount Sinai Hospital New York New York USA; ^7^ Schmidt College of Medicine Florida Atlantic University Baca Raton Florida USA; ^8^ University of Florida College of Medicine Gainesville Florida USA; ^9^ Dermatology of Boca Baca Raton Florida USA; ^10^ The Dermatology Institute of Boston Boston Massachusetts USA; ^11^ Northeastern University Boston Massachusetts USA

**Keywords:** 1726 nm laser, acne, contact cooling, laser therapy, sebaceous glands, selective photothermolysis, severe acne

## 1 Introduction

The contact‐cooled 1726 nm laser (Aviclear, Cutera Inc., Brisbane, CA) treats acne vulgaris by selective thermolysis of sebaceous glands [1]. The device has demonstrated efficacy and safety across a broad range of patients with mild to severe inflammatory acne in clinical trials [2, 3]. In the pivotal trial leading to FDA clearance, 104 subjects (103/104 were graded as moderate or severe at baseline) received three treatments at 2–5 week intervals [3]. A per‐protocol analysis of the trial (Table 1) showed significant improvements in both inflammatory lesion count (ILC) and investigator global assessment (IGA) [3, 4]. While study findings are promising, guidance on effective translation of findings to real‐world clinical practice and how to best approach patients who may already be on medications is needed.

**TABLE 1:** Clinical study outcomes for the contact‐cooled 1726 nm laser at 3 months and 1 year.

	3 months (%)	1 year (%)
Primary endpoint		
Responder rate (% with ≥ 50% ILC reduction)	79.8	91.5
Secondary endpoints		
IGA improvement (% clear/almost clear)	35.9	66.2
ILC reduction (median)	55.9	78.6
Non‐inflammatory lesion count reduction (median)	25.0	57.6

## 2 Methods

Here, an expert consensus group of five dermatologists presents best‐practice recommendations for patient selection, treatment optimization, adjunctive therapies, and management strategies. Panelists were selected based on having performed the highest number of contact‐cooled 1726 nm treatments, to date, as well as recognized expertise in managing acne. A nominal group technique was used: individual participant best practices were first collected individually via 1‐h phone interview and clarified via follow‐up e‐mail communications. Next, a series of two 1‐h virtual discussions were held, where panelist approaches were shared and discussed and a consensus was developed on the guidance for clinical practice presented here. The recommendations presented herein have been approved by all panelists/authors. Agreement was discussion based and there was no formal voting or Delphi process. Recommendations are based on clinical experience in treating over 1600 patients, collectively.

## 3 Results

### 3.1 Ideal Candidates for 1726 nm Laser Treatment

Contact‐cooled 1726 nm laser treatment is appropriate for patients in puberty or older with inflammatory acne, regardless of skin type. Ideal candidates typically have moderate to severe inflammatory acne, as improvement is most notable in this population; however, use is appropriate in patients with mild acne who decline other treatment options. While treatment with contact‐cooled 1726 nm laser can be effective as monotherapy (with or without systemic medications), in the authors' experience, most patients benefit from combination regimens that include topical and/or oral therapies.

Patients with mild or comedonal acne should be managed according to the American Academy of Dermatology (AAD) treatment guidelines [5]—beginning with topical therapy and, if needed, oral agents. Contact‐cooled 1726 nm laser treatment may be introduced if inflammatory acne persists beyond 3 months of standard care or if the patient desires a non‐pharmaceutical treatment.

Treatment‐experienced patients, including those who have failed prior therapies, may still respond to treatment with contact‐cooled 1726 nm laser treatment; however, realistic expectation‐setting is crucial, particularly for more severe patients with a history of isotretinoin use. In the authors' clinical experience, partial responders to isotretinoin (e.g., those who did not complete a full course or those whose acne recurred after past courses of isotretinoin) may see improvement with laser treatment, though outcomes can vary.

### 3.2 Predictors of Treatment Response

**FIGURE 1:**
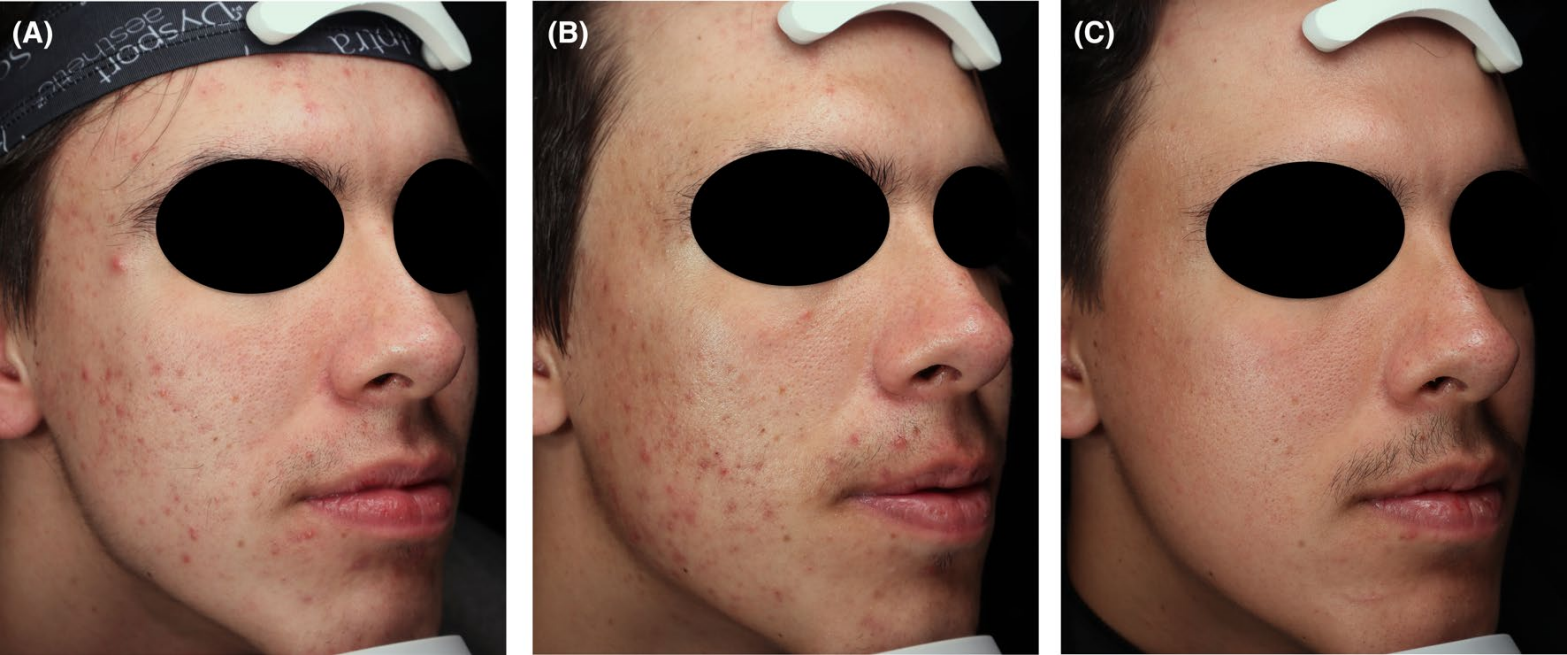
An 18‐year‐old male at baseline (A) and 1 month (B) and 6 months (C) after three treatments with 1726 nm contact‐cooled laser. Treatment parameters were 18 J/cm^2^ (300 pulses) for treatment 1, 21 J/cm^2^ (300 pulses) for treatment 2 and 22.5 J/cm^2^ (300 pulses) for Treatment 3, administered 4 weeks apart. The patient was also treated with Excel V+ (532 nm, 7.2 J/cm^2^, 10 mm, 10 ms, 5 C) and a Vivier Jessner Chemical Peel 2 weeks after the third 1726 nm treatment and every 3 months thereafter for 1 year and used topical skincare (SkinMedica AHA/BHA cleanser and cream).

At present, no definitive predictors of response to contact‐cooled 1726 nm laser treatment exist. However, clinical experience suggests better outcomes in patients who adhere to a comprehensive regimen that includes adjunctive topical or oral acne treatments and appropriate skincare. Among the five surveyed practices included in the panel, adult females, who comprise 67% of treated patients in expert practices, appear to respond most consistently. The most notable improvements are generally seen in those with at least moderate inflammatory acne. An example of a patient managed with combination therapy is shown in Figure 1.

### 3.3 Setting Patient Expectations

Clear and transparent communication is vital to managing expectations. Patients should be informed of the following features of contact‐cooled 1726 nm laser treatment:

- Results are not immediate. Final improvement is typically seen 3 to 6 months after the last treatment. This observation is consistent with the pivotal trial, where inflammatory lesion counts continued to reduce over time.
- Treatment discomfort and transient acne flares are possible.
- An acne flare after each or any treatment is common and should not discourage continuation.
- Education about acne pathophysiology, treatment timelines, and the importance of ongoing skincare regimens—including during and after contact‐cooled 1726 nm laser treatment sessions helps to maintain compliance and patient satisfaction.

### 3.4 Treatment Protocol and Settings

Optimal treatment typically involves 250 to 300 pulses per session, as per on‐label treatment guidelines. Early in the launch phase of AviClear, the pulse count range for the authors was 160–252. After manufacturer treatment guidelines were updated in March of 2024, pulse counts were increased accordingly. In the authors' experience, better clearance is obtained in patients who are treated with as close to the 300 pulses as possible. The recommended approach includes:

- Global facial treatment in the first pass.
- Second pass over problem areas using remaining pulses.
- Cooling time between passes to enhance comfort and safety.

The average fluence used by the authors is 20 J/cm^2^, with starting fluence at 19.5 J/cm^2^, titrated upward based on patient tolerability, as determined at the first treatment visit. Once patient tolerability is established, fluence is incrementally increased at subsequent visits. Higher fluence may also be achievable by using a water‐based topical lidocaine cream to increase patient comfort during the procedure. Any oil‐containing numbing agents should be avoided: the laser itself is specific to oil and inadequate degreasing can contribute to burns and interfere with contact‐cooled 1726 nm laser treatment [6].

### 3.5 Additional Treatments and Management of Nonresponders

In the pivotal trial, 8.5% of patients were classified as nonresponders, defined as achieving less than 50% improvement at 52 weeks [4]. In our real‐world experience, approximately 10% of patients receiving three sessions of treatment with the contact‐cooled 1726 nm laser can be considered partial responders, and demonstrate some degree of improvement, yet fail to meet the 50% improvement threshold used in the clinical trial. True nonresponders in the clinical setting—those showing no change from baseline or worsening—account for roughly 5% of cases. In the author's experience, many patients who can be considered partial responders, or who wish to achieve greater clearance, can achieve better results with additional contact‐cooled 1726 nm laser treatments. The following are experience‐based recommendations for patient follow‐up and considerations for administering additional treatments:

- Delay evaluation of final treatment response until ≥ 4 months after the third session.
- Offer a fourth treatment for:
  - Patients with partial but ongoing improvement.
  - Patients who initially improved but have plateaued or regressed (booster treatment).
  - Patients with minimal improvement who wish to avoid systemic therapies but remain satisfied with 1726 nm laser treatment.
- At follow‐up, assess adherence to topical medications and skincare regimens.
- For patients determined to be nonresponders (worsening from baseline or no improvement at any time point), alternative therapeutic options should be explored.

### 3.6 Use of Adjunct Medications and Devices

In the contact‐cooled 1726 nm laser clinical trials, adjunct medications and devices were not permitted. While this allows for quantification of response to contact‐cooled 1726 nm laser treatment, in real‐world clinical practice, patients often receive multiple interventions. While effective, combination treatment can confound assessment of laser‐specific efficacy. In clinical practice, staging of treatments can inform the impact of individual treatments. Below are experience‐based recommendations for concomitant therapy.

#### 3.6.1 Topical Agents

Topical retinoids [7, 8] (alone or in combination with benzoyl peroxide or antibiotics) are recommended for all patients, particularly those with comedonal acne. Initiation should ideally occur 1 month before contact‐cooled 1726 nm laser treatment to acclimate the skin and optimize results.

#### 3.6.2 Hormonal Therapies

For females with hormonal acne, spironolactone [8] possibly along with oral contraceptive pills and topical clascoterone may be initiated before, during, and after contact‐cooled 1726 nm laser therapy.

#### 3.6.3 Energy‐Based Devices and Cosmetic Procedures

In the authors' clinical experience, effective adjunctive treatments can include:

- Exfoliating facials, superficial chemical peels, or microdermabrasion to manage comedonal acne.
- 1064 nm Nd:YAG lasers to reduce bacterial colonization and inflammation [9], particularly in adult females.
- A vascular laser to target post‐inflammatory erythema and give possible improvement in active acne [10].

### 3.7 Oral Medications for Flare Prevention and Management

Acne flares are common during contact‐cooled 1726 nm laser therapy. In the users' experience, the rate of flares in real‐world practice is considerably lower (20%) than that reported in the pivotal study, where 45% of patients experienced flares after at least one treatment [3]. This side effect is also seen with isotretinoin usage [11] and should not raise major concerns. Oral antihistamines, known to mitigate isotretinoin‐related flares [12], may provide similar benefits for treating and preventing flares with contact‐cooled 1726 nm laser; however, there are no data on use in this setting (Table 2). Proper patient counseling regarding the possibility of an acne flare is critical to maintain patient compliance with all three contact‐cooled 1726 nm laser treatments.

**TABLE 2:** Recommendations for managing common concomitant medications.

Medication	Dosage	Duration
Spironolactone	50–200 mg PO daily	Entire treatment course
Oral antihistamines	Per package insert	Entire treatment course
Sarecycline	QD (weight‐based)	Entire treatment course
Doxycycline	100 mg PO BID	Up to entire treatment course

### 3.8 Other Possible Adverse Events

In the authors' experience, contact‐cooled 1726 nm laser treatment is well tolerated and the safety profile of the study treatment is consistent with clinical experience thus far. In clinical studies, treatment had an average pain score of 5.2 out of 10, with no pain management [2]. Temporary erythema and edema was observed in all patients (all cases were resolved by week 12) and there were no instances of residual erythema, edema, blistering, crusting hypo, or hyperpigmentation occurred. Mild dryness was observed in 44.2% of patients; however, in the study and in clinical practice, this is easily managed with moisturizer [2]. Of note, the contact‐cooled 1726 nm laser is the first laser shown to be effective and with a favorable tolerability profile in all skin types II through VI.

### 3.9 Posttreatment Regimen

After completing contact‐cooled 1726 nm laser therapy treatment, patients should continue pre‐treatment topical regimens, including retinoids, moisturizers, and sunscreen as well as oral regimens. If a retinoid was not previously used, it should be introduced to maintain results and prevent recurrence. Once clear skin and/or patient satisfaction is achieved, other adjunctive therapies may be discontinued. In some cases, topical or oral therapeutics may be continued. Contact‐cooled 1726 nm laser therapy results have been shown to persist up to 1 year following treatment (Table 1) [13].

## 4 Conclusion

Contact‐cooled 1726 nm laser therapy represents an innovative and effective addition to the acne treatment armamentarium. Its targeted selectivity for sebaceous glands and sustained clinical results set it apart from earlier laser‐based modalities. Achieving optimal outcomes requires careful patient selection, clear expectation‐setting, thorough counseling regarding the potential for transient acne flares, and the judicious use of adjunctive medications and supportive therapies. As clinical experience and evidence continue to accumulate, treatment protocols will likely become increasingly individualized, further refining and enhancing the role of contact‐cooled 1726 nm laser therapy in comprehensive acne management.

## Author Contributions

All of the Authors Listed Participated in the Development of Recommendations and Each Aided in the Development and Review of This Commentary.

## Ethics Statement

The authors have nothing to report.

## Consent

The patient featured provided written consent for use of their image.

## Conflicts of Interest

All authors serve as consultants for Cutera Inc.

## Data Availability

Data sharing not applicable to this article as no datasets were generated or analyzed during the current study.
